# Out-of-hospital cardiac arrest in three EMS centers in northern and central Thailand: a mixed-methods call for a national data infrastructure

**DOI:** 10.1186/s12873-026-01629-1

**Published:** 2026-05-29

**Authors:** Chanodom Piankusol, Nutcha Charoenboon, Natthanaphop Isaradech, Borwon Wittayachamnankul, Sattha Riyapan, Jirapong Supasaovapak, Robert Bonar, Bryan McNally, Wachiranun Sirikul

**Affiliations:** 1 Milken Institute School of Public Health, George Washington University, Washington D.C., USA; 2 Department of Community Medicine, Faculty of Medicine, Chiang Mai University, 110 Intawaroros Road, Sriphum, Mueang, Chiang Mai, 50200 Thailand; 3 Environmental and Occupational Medicine Excellence Center, Faculty of Medicine, Chiang Mai University, Chiang Mai, Thailand; 4 Department of Emergency Medicine, Faculty of Medicine, Chiang Mai University, Chiang Mai, Thailand; 5 Department of Emergency Medicine, Faculty of Medicine Siriraj Hospital, Mahidol University, Bangkok, Thailand; 6 Rajavithi Hospital, Ministry of Public Health, Bangkok, Thailand; 7 Emory University School of Medicine, Emory University, Atlanta, USA; 8 Center of Data Analytics and Knowledge Synthesis for Health Care, Faculty of Medicine, Chiang Mai University, Chiang Mai, 50200 Thailand

**Keywords:** Out-of-hospital cardiac arrest, OHCA registry, emergency medical services, EMS systems, Neurological outcomes, Thailand, Health data systems, Mixed methods, Health policy, low- and middle-income countries

## Abstract

**Background:**

Out-of-hospital cardiac arrest (OHCA) is a leading cause of mortality in Thailand, yet national response to the issue remains fragmented and under-prioritized. There is no centralized OHCA registry, neurological outcome data are almost entirely absent, and coordination between EMS and hospitals is weak. This limits Thailand’s ability to evaluate performance, guide investments, or improve survival.

**Methods:**

We conducted a convergent mixed-methods study combining retrospective analysis of OHCA registry data (*n* = 2,259) from three hospital sites participating in a regional OHCA registry (2017–2023) with in-depth qualitative interviews across EMS, hospital, and policy stakeholders. Quantitative data were analyzed for trends in bystander CPR, response and scene times, and outcome tracking. Qualitative data were thematically coded to identify structural and operational barriers to data use and system integration.

**Results:**

Neurological outcome data were available for only 16.4% of patients who were alive at 30 days. EMS teams had limited access to in-hospital outcome information, and hospitals did not routinely track functional recovery. One suburban-capital hospital showed measurable improvement using internal data to reduce scene times and increase bystander CPR. However, systemic fragmentation undermined scalability. Across sites, EMS and hospital data systems were not interoperable, and OHCA care was inconsistently prioritized.

**Conclusion:**

Thailand’s OHCA system shows both promise and constraint. These findings highlight the need for improved data infrastructure, including standardized outcome tracking, better linkage between EMS and hospital systems, and clearer governance. Local successes can serve as models, but only with political will, shared infrastructure, and clear governance. A national OHCA data strategy is essential to improve survival and achieve equitable care.

**Supplementary Information:**

The online version contains supplementary material available at 10.1186/s12873-026-01629-1.

## Introduction

Out-of-hospital cardiac arrest (OHCA) is a leading cause of global mortality, affecting hundreds of thousands annually [1]. In many high-income countries, coordinated emergency medical systems (EMS) and national registries have led to substantial gains in survival and neurological outcomes following OHCA [2]. Examples from countries such as South Korea, Japan, Singapore, and Sweden have shown that when data are collected and used systematically, interventions such as bystander CPR, timely defibrillation, and targeted post-resuscitation care can dramatically improve patient outcomes [3, 4]. These systems benefit from unified dispatch, consistent training standards, and government investment in performance monitoring, often tied to national or regional OHCA registries. These registries enable real-time analysis of EMS performance, hospital-level care, and longer-term patient outcomes, including neurological function and return to community [5–7].

In contrast, many low- and middle-income countries (LMICs) remain without even basic mechanisms to track OHCA events, let alone monitor interventions or outcomes [8, 9]. Thailand exemplifies this gap. Although EMS systems have expanded rapidly since the early 2000s, particularly in trauma response and general emergency preparedness, OHCA remains under-prioritized and poorly documented. There is no nationally mandated data reporting system for OHCA, no formal protocol for neurological outcome monitoring, and minimal interoperability between pre-hospital and hospital care teams. As a result, the burden of OHCA is difficult to quantify, and efforts to improve survival are inconsistently applied [10–12].

Multiple factors contribute to this gap. First, OHCA has yet to receive significant global prioritization. Unlike other time-sensitive conditions, such as maternal mortality, road traffic injury, or stroke, OHCA is not a core indicator in most global health development frameworks [13]. The Sustainable Development Goals (SDGs), for instance, include targets related to universal health coverage and road traffic deaths but make no mention of cardiac arrest, despite its prevalence [14].

Second, Thailand’s EMS system is structurally fragmented. While the Ministry of Public Health (MoPH) oversees advanced life support (ALS) units, many basic life support (BLS) teams are under the jurisdiction of the Ministry of Defense or local municipalities. Dispatch centers vary by region, and there is no uniform national protocol for EMS call triage, handoff, or documentation. These inconsistencies directly affect the country’s ability to respond efficiently to OHCA and to learn from cases that do occur [15].

Third, the country lacks mechanisms for feedback and continuous improvement in resuscitation care. In many settings, EMS teams do not know whether their patients survived, whether CPR was effective, or whether transport decisions were appropriate. Neurological status post-resuscitation is rarely documented. This absence of feedback limits motivation, weakens quality improvement loops, and undermines the potential for data-driven change [16, 17].

Despite these challenges, there are bright spots. The suburban-capital site highlighted in this study have built internal systems for OHCA tracking and quality improvement. These systems are often led by motivated clinicians and rely on dedicated staff to maintain records and convene weekly case reviews. Though not formally integrated into national policy, they provide proof of concept that data-informed care is both feasible and impactful.

This paper presents a mixed-methods analysis of OHCA care in Thailand, drawing from registry data and qualitative interviews with EMS providers, hospital staff, and policymakers. It highlights the structural and cultural barriers to data collection and system integration, while offering actionable lessons from one high-performing local system. The central argument is that Thailand needs a national OHCA data infrastructure, not only to improve individual outcomes but to coordinate learning, align incentives, and elevate OHCA as a policy priority. This is not a call for importing expensive technology, but for implementing basic, scalable, and context-sensitive mechanisms for information sharing and accountability.

This study draws on data from three PAROS-participating hospital systems and is not intended to provide a nationally representative assessment. However, these sites represent large, well-established public hospitals with relatively advanced EMS capabilities and participation in international data networks. As such, they offer important insight into system-level challenges and opportunities for improvement within the Thai OHCA context. Findings from these settings may therefore help identify priority areas for strengthening data systems and coordination more broadly.

## Methods

This study employed a convergent parallel mixed-methods design, in which quantitative and qualitative data were collected and analyzed concurrently and integrated at the interpretation stage [18]. This approach enabled quantitative trends in out-of-hospital cardiac arrest (OHCA) care to be contextualized through stakeholder experiences and system-level insights.

### Study setting

Thailand operates a multi-tiered emergency medical services (EMS) system comprising both Basic Life Support (BLS) and Advanced Life Support (ALS) teams. ALS teams are generally overseen by the Ministry of Public Health (MoPH) and are based in larger hospitals, while BLS teams may be funded and managed by local administrative organizations or the Ministry of Defense, particularly in rural areas. Dispatch protocols vary widely between regions, and there is currently no unified national electronic health record system linking EMS and in-hospital care.

EMS personnel in Thailand include a mix of physicians, nurses, paramedics, and trained volunteers, with varying levels of formal education and certification depending on the service level and managing organization. Training standards and operational capacity differ across regions, particularly between urban and rural areas. While major urban centers typically have more consistent access to ALS services, rural areas may rely more heavily on volunteer-based BLS units with longer response times and more limited resources. These variations influence both service delivery and data collection practices across the EMS system.

Three hospital systems were selected for this study to reflect geographic and institutional diversity: a regional referral hospital in northern Thailand, an urban-capital hospital in the Bangkok metro area, and a suburban-capital hospital with a reputation for EMS innovation. These sites also participated in the Pan-Asian Resuscitation Outcomes Study (PAROS) network during the study period (2017–2023), providing a shared data infrastructure for the quantitative analysis.

PAROS is a multinational registry that collects standardized data on OHCA across participating sites in Asia using Utstein-style reporting templates, including patient characteristics, arrest circumstances, pre-hospital interventions, and outcomes. Participation in PAROS is voluntary, and in Thailand, only a limited number of hospitals contribute data, primarily due to differences in local capacity, data infrastructure, and institutional engagement. As a result, most EMS systems in Thailand are not currently represented within this registry.

### Quantitative data and analysis

Quantitative data were obtained from the PAROS registry for the three study sites, covering the period 2017–2023. Inclusion criteria were adult OHCA cases (age ≥ 18) attended by EMS teams. Data collection followed Utstein-style reporting guidelines for OHCA, enabling standardized comparison across sites [19]. Variables included patient demographics, arrest characteristics (e.g. witnessed status, initial rhythm), EMS response and scene times, pre-hospital interventions (e.g. bystander CPR, AED use), and clinical outcomes including return of spontaneous circulation (ROSC) and survival.

Neurological outcomes were assessed using the Cerebral Performance Category (CPC) scale [20], a widely used measure ranging from good cerebral performance (CPC 1) to severe disability or coma (CPC 4–5). However, as described in the Results, these data were largely incomplete across sites.

Descriptive analyses were conducted using Stata/MP 17.0 (StataCorp. 2025. *Stata Statistical Software: Release 17*. College Station, TX: StataCorp LLC.). Trends over time and differences between sites were examined using non-parametric tests where appropriate. Data completeness and quality were also assessed to reflect variability in registry implementation.

### Qualitative data and analysis

The qualitative component consisted of 23 in-depth interviews with EMS providers, emergency physicians, hospital administrators, policymakers and the MoPH, and data managers involved in OHCA tracking. Participants were purposively sampled to capture a range of experiences and institutional roles and perspectives. Interviews were conducted in Thai using a semi-structured guide exploring five domains: EMS operations, data practices, inter-agency collaboration, barriers to OHCA care, and policy suggestions.

Audio recordings were transcribed verbatim, translated into English, and uploaded to NVivo 12 for coding. The coding framework was developed both inductively and deductively: initial codes were derived from interview questions and health systems literature, while emergent codes were added through iterative reading. Key themes included registry implementation challenges, differences between service arms, variation in local policies, the role of champions in driving change, and ideas for national reform. Triangulation with quantitative findings allowed for validation and contextualization of performance patterns and exploration of causal mechanisms. Integration of quantitative and qualitative findings occurred during interpretation, where qualitative data were used to explain patterns observed in registry data, particularly regarding data gaps, system fragmentation, and variation in practice across sites.

### Ethics and rigor

Ethical approval for the study was obtained from Chiang Mai University’s Research Ethics Committee (AF/04–009/02.0, Study code: COM-2567-0607). All interview participants provided written informed consent prior to participation. Data were de-identified before analysis to ensure confidentiality. For the quantitative component, the requirement for informed consent was waived as the study involved secondary analysis of de-identified registry data. To ensure analytical rigor, double coding was performed on a subset of transcripts, and discrepancies were resolved through consensus discussions among the research team. Field notes and contextual memos were used to supplement transcript data and preserve cultural nuance during translation. All research procedures adhered to the Declaration of Helsinki and relevant national guidelines.

## Results

Three central findings emerged from the mixed-methods analysis: (1) neurological outcomes were rarely recorded and OHCA data remained largely invisible in the national EMS system, (2) one suburban-capital hospital demonstrated tangible improvements through local data use, and (3) systemic fragmentation across institutions, data systems, and service cultures was the most consistent barrier to OHCA care advancement. These themes were supported by registry data trends and deeply echoed in qualitative interviews across all three sites. Detailed patient eligibility and characteristics are presented in the supplementary materials (Supplementary Fig. 1 and Supplementary Table 1).

### National blind spots in neurological and outcome data

From Table 1, neurological outcome data were recorded in only 11 of 67 patients who were alive at 30 days (16.4%) across all three sites, highlighting substantial gaps in post-resuscitation follow-up (see Supplementary Table 2 for detailed CPC and OPC data).

**Table 1:** Recorded neurological outcome data in three hospitals through the study period

Patient Characteristics	Thailand OHCA Data Collection Centers (Total = 2259)						*P*-value
	Regional hospital (2017–2023) (*n* = 569)		Urban-capital hospital (2017–2020) (*n* = 785)		Suburban-capital hospital (2017–2023) (*n* = 905)		
	*N*	(%)	*N*	(%)	*N*	(%)	
Patient Neurological Status							
Collected	0	0.00	1	0.13	10	1.10	< 0.001
Not Collected	569	100	784	99.87	895	98.90	
30-day Post-arrest Hospital Outcome							
Alive	36	6.33	12	1.53	19	2.1	< 0.001
Death	533	93.67	773	98.47	886	97.9	

Abbreviations: Significant differences from post-hoc pairwise comparisons (*p* < 0.05) using Bonferroni method: a regional vs. urban-capital, ^b^ urban-capital vs. suburban-capital, ^c^ suburban-capital vs. regional

As shown in Table 2, interviewees described this not as a technical glitch, but as a structural limitation. There was no mandate, formal workflow, or incentive for post-resuscitation follow-up or cross-departmental data sharing. Hospital units often did not consider OHCA patients admitted via EMS as a unique cohort requiring tracking or longitudinal evaluation. As a result, data officers reported limited familiarity with the CPC scale, and neurological outcome data – when documented – were often embedded in unstructured, sometimes paper-based clinical notes that were not routinely captured in registry systems. (Original Thai-language quotes corresponding to the English excerpts presented in the tables and text are provided in Supplementary Appendix 1).

**Table 2:** Quotes illustrating structural barriers to post-resuscitation outcome tracking and EMS–hospital data sharing

Quotes	Interviewees
We have little data on neurological outcomes because there aren’t many cases that survive. A lot of patients are dead on scene, at the ER, or in the hospital. Not many survive until discharge. If I remember correctly, survivors only make up 6–7% at our [urban-capital] hospital, and those who survive with good neurological outcome is a subset of this group, so we barely see them. When we export PAROS data, this group is really small.	EMS Supervisor, Urban Hospital
We aren’t rigorous with collecting neurological [outcome] data; we don’t call patients to interview after they’ve been discharged. Someone had made a suggestion, too, to start collecting these data because there’re less than 10 patients who can go home alive per year. We have a set of questions for these survivors, but we don’t ask them these questions formally. It would take close to half an hour to collect all those data, it’s complicated. If we wanted to stick to the book, we should make an appointment to do a follow-up with them. Like in Singapore, they even organize a Survivor Club (…) But I have to admit that in Thailand, we can’t focus on quality of life yet, we must prioritize just survival first [regardless of quality].	Emergency Physician, Suburban Hospital

Compounding this issue was a disconnection between EMS and in-hospital care. Most EMS teams had no access to hospital records, and even in cases of ROSC, were unable to confirm patient outcomes beyond ER admission. Where follow-up occurred, it was reliant on information channels such as personal contacts or backchannel communications between departments. This lack of visibility not only impeded system learning but also reduced morale among EMS providers, who felt that their efforts were detached from final patient outcomes.

While the absence of systematic outcome data was widely described as a structural limitation, participants differed in how they navigated this gap in practice. Some described data systems as fragmented and burdensome, whereas others highlighted efforts to derive meaningful clinical insights from available records, particularly regarding patients’ neurological recovery.

We may receive the medical record (…) at times we have to read and interpret it ourselves to determine whether the patient was awake, regained consciousness, or able to follow commands. These are often nursing notes that we have to decipher.

EMS Supervisor, Urban Capital Hospital

These accounts suggest that, even in the absence of standardized measures such as CPC, clinicians recognize the importance of neurological outcomes and attempt to reconstruct them from available clinical documentation.

### Measurable progress at the suburban-capital hospital

**Fig. 1:**
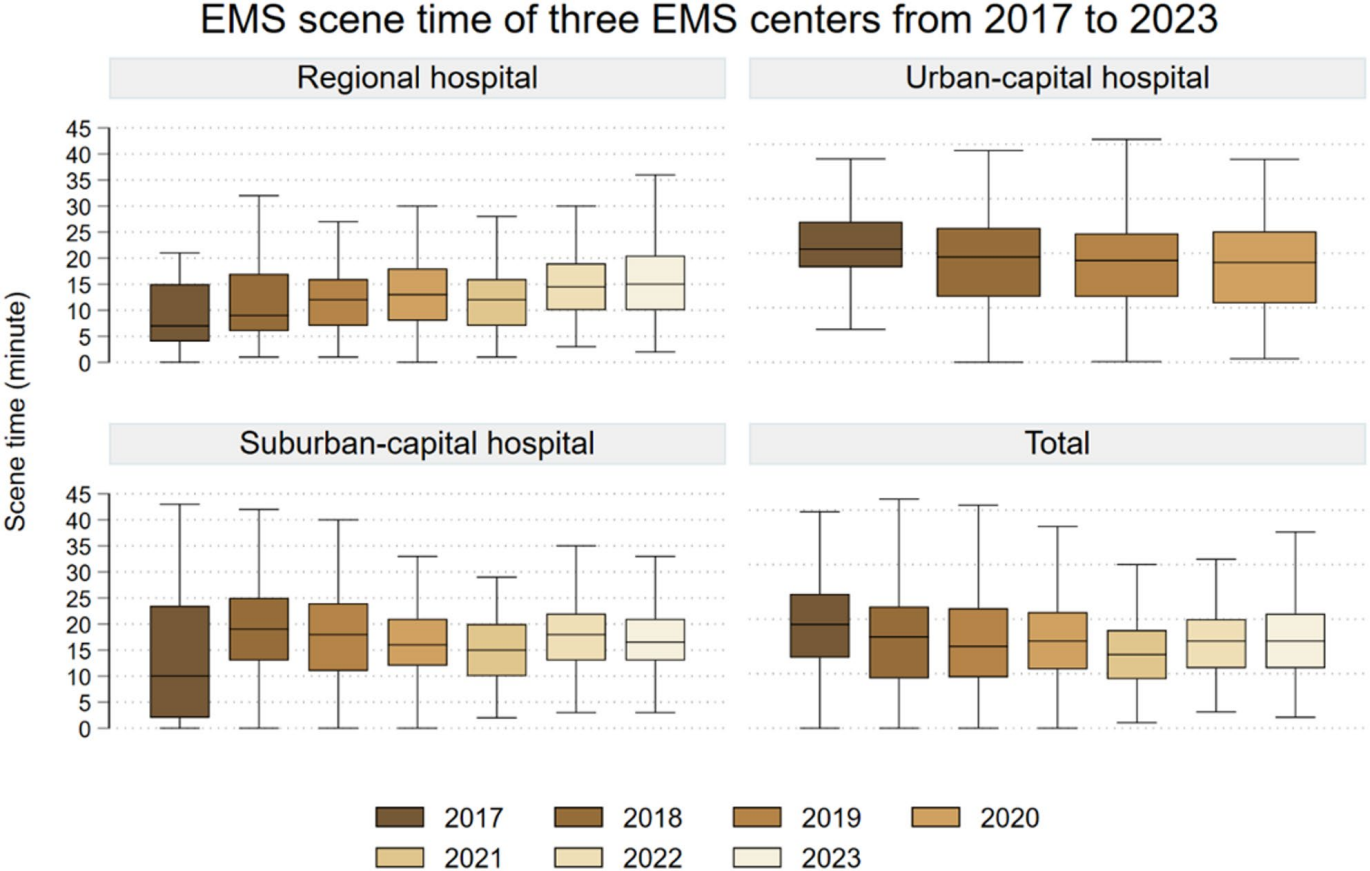
Scene times of three EMS centers from 2017 to 2023

Amidst these challenges, one hospital demonstrated marked improvements over the six-year period. This site had built an internal registry and quality improvement program led by a multidisciplinary team. Key indicators showed upward trends: bystander CPR increased from 25% to over 51%, scene time decreased year-on-year, and AEDs were deployed more frequently in high-incidence locations as shown in Fig. 1.

The hospital implemented weekly case review meetings, telemedicine support during high-priority calls, and a geospatial mapping system for dispatch optimization. These interventions were guided directly by local data, which were cleaned and validated weekly by an EMS data officer. Hospital leadership supported protected time for EMS quality improvement work and used registry reports to advocate for new AED placements and CPR training campaigns in surrounding communities (Table 3).

**Table 3:** Quotes illustrating local data-driven quality improvement practices in a suburban-capital hospital

Quotes	Interviewees
Back then, our bystander CPR rate was only 20%, that’s why we tackled the community CPR challenge. Right now we’re up to 50% with quite a large [positive] impact. Since then, we continue to develop other projects. All of them started from having data.	Emergency physician, Suburban-Capital Hospital
We gained information from [OHCA] registry and we analyzed to see which areas were missing what. Then we adjusted the locations of AED.	Hospital Admin, Suburban-Capital Hospital
We have a meeting every week to see if our dispatch units are slow or whether there’s anything they should improve on.	EMS Supervisor, Suburban-Capital Hospital
We use telemedicine to follow and record the data better.	Data Coordinator, Suburban Hospital
The registry is the foundation of everything. When we started to carry out the registry, we found that our problem was that survival rate wasn’t good. So we initiated many interventions, and we then followed [their results] using the registry.	EMS Supervisor, Suburban-Capital Hospital

Participants from this site emphasized that data was not only used for reporting but for iterative decision-making. Dispatchers received feedback on triage speed, and on-scene paramedics discussed response time anomalies in team meetings. This dynamic created a culture of accountability and learning rarely seen in other sites. Importantly, these achievements were not attributed to extra resources, but to alignment between leadership, policy, and operational staff.

### Structural fragmentation and policy incoherence

Despite isolated pockets of innovation, the most common theme across all interviews was fragmentation. EMS in Thailand is split between the Ministry of Public Health (MoPH) and the Ministry of Defense (MoD), with local governments also managing certain units. This has resulted in multiple dispatch numbers, differing training standards, and incompatible data systems. Several interviewees described scene conflicts where BLS and ALS teams operated under different protocols or mistrusted each other’s clinical judgment (Table 4).

**Table 4:** Quotes illustrating system fragmentation across EMS agencies and clinical protocols

Quotes	Interviewees
The staff members of the service [EMS] units and of the emergency call centres don’t trust one another.	EMS Leader, Urban Hospital
The ALS team will not leave their base if the BLS team hasn’t confirmed that the case is real [qualified for Advanced aid] yet.	Dispatcher, Urban Hospital
Each place [hospital/dispatch centre] has their own system. Some places don’t approve the BLS team to move the patient if that BLS team isn’t of their own network.	BLS Team Member, Regional Hospital
When they arrive at the scene, sometimes the BLS and ALS teams would keep passing the buck about who should be responsible [for the patient].	ALS Paramedic, Urban Hospital

This fragmentation extended to data entry responsibilities. At some sites, paramedics were expected to complete registry forms during transport or immediately after ER handover, tasks often skipped due to time pressure. At others, administrative staff unfamiliar with clinical events handled data cleaning, leading to errors and missing fields. Data collection was frequently perceived as bureaucratic rather than clinical (Table 5).

**Table 5:** Quotes illustrating inconsistent data entry practices and barriers to accurate OHCA documentation

Quotes	Interviewees
We can only keep track [of the data] within the ER service. When the patient is admitted as an in-patient, we cannot keep following their data.	Data Officer, Regional Hospital
We don’t have the system that automatically connects the EMS [data pool] with the hospital.	IT/Registry Staff, Suburban Hospital
The data that the EMS team records don’t get used within the hospital.	Hospital Admin, Urban Hospital
Some inpatient charts aren’t 100% electronic, sometimes we have to ask the record department for specific data to do a case review. So that’s a limitation. Our clearance to access data from the in-patient departments isn’t high. The front-line workers [EMS personnels] do not have the clearance to view treatment records until discharge. In some cases, we might obtain a discharge summary. But it all depends on luck whether we get a detailed discharge summary or not.	Emergency Physician, Urban Hospital
Sometimes we don’t have anyone to follow up for evaluation, except during periods when we have someone collecting data for us very frequently. In this case, we can still gain an understanding of the patient’s condition during the 1–2 weeks they are admitted. We can still go and monitor them in the ward, but if it’s a 3-month follow-up, we don’t see anything. (…) It needs to connect pre-hospital data with emergency [ER] data and then with in-patient data in the hospital. If there is a large team, it is possible to follow up on this data point.	Emergency Physician, Urban Hospital

Furthermore, regional policy autonomy meant that hospitals could develop their own protocols, dispatch priorities, and response targets without reference to national benchmarks. Without centralized governance, successful local models like the suburban-capital hospital remained isolated examples rather than scalable innovations (Table 6).

**Table 6:** Quotes illustrating variability in regional OHCA policies and the absence of national governance

Quotes	Interviewees
The Erawan Centre [Bangkok Emergency Medical Centre] is a local administrative organization’s unit. (…) Their policy changes according to their leaders.	Hospital Admin, Urban Hospital
There’s no agency that’s directly and clearly responsible for the OHCA patients.	NIEMS Official
The policies differ in every area. Some dispatch units spend a lot of time before they commit [to cases].	Dispatcher, Regional Hospital
The systems in Bangkok and in provinces outside of Bangkok are completely different. It’s difficult to compile all the data.	IT Admin, Urban Hospital

Abbreviation: NIEMS, National Institute for Emergency Medicine

Although structural fragmentation and limited institutional ownership were consistently reported, participants differed in how they interpreted the feasibility of change. Some emphasized systemic inertia and competing priorities within the health system, while others described opportunities to leverage political windows, external agendas, or leadership responsiveness to advance OHCA-related initiatives.

If there is public momentum, senior leadership may suddenly request information in order to respond to the media, often requiring a response within one day.

Health Policy Advisor

These accounts suggest that, while structural barriers persist, pathways for change may emerge when initiatives align with broader attention, leadership priorities, and external pressures.

This study reveals both the potential and the limitations of OHCA care in Thailand. Through an in-depth examination of EMS registry data and extensive interviews with frontline providers and policymakers, three critical insights emerge. First, Thailand lacks the fundamental infrastructure to track patient outcomes following OHCA, particularly neurological recovery. Second, local success is possible, even in resource-constrained settings, when data are used systematically to inform practice. Third, structural fragmentation across ministries, agencies, and protocols inhibits the national coordination required to improve OHCA outcomes at scale. Together, these findings highlight the urgent need for a national OHCA registry, embedded in an interoperable EMS system, with clear governance and real accountability mechanisms.

## Discussion

### The importance of outcome visibility

Without data on neurological outcomes, it is impossible to know whether resuscitation efforts are achieving meaningful success. The absence of this information undermines both quality improvement and policy advocacy. In Thailand, where most hospitals do not track functional status post-ER, resuscitation success is often defined as “arrival with pulse” rather than survival to discharge or return to community. This is not merely a technical gap; it reflects a misalignment between what systems record and what patients and families value.

Neurological outcome monitoring is a global standard in OHCA research and system design [20]. Tools such as the Cerebral Performance Category (CPC) scale and structured hospital follow-up protocols allow for benchmarking, longitudinal improvement, and resource prioritization. The PAROS network, of which Thailand is a member, encourages these practices, yet national-level implementation remains elusive. Interviewees in our study described how missing outcome data weakened their ability to secure funding, demonstrate progress, or motivate EMS teams. In the absence of feedback loops, even successful resuscitations feel incomplete.

Moreover, without outcome data, governments cannot compare sites, allocate resources strategically, or identify equity gaps. As OHCA cases increase alongside urbanization and population aging, Thailand risks widening the performance gap between hospitals that invest in data and those that do not [21]. A national outcome tracking system is not simply a research tool, but it is a health system necessity.

### Lessons from local innovation

The suburban-capital hospital studied here provides a powerful case for what is possible within existing constraints. Without external mandates, this site improved multiple performance indicators using internal data, consistent leadership, and protected time for quality improvement. Staff at this hospital used registry trends to redesign dispatch algorithms, target community CPR training, and advocate for AED placement. Importantly, this success was made possible by institutional alignment: administrators, EMS leaders, and clinical staff shared a vision and coordinated efforts.

This model aligns with broader evidence from other LMICs. Studies in India and Malaysia have shown that local EMS teams, when given autonomy and analytical tools, can identify delays and reduce scene times [22, 23]. South Korea’s transition to national OHCA reporting began with pilot projects in select cities [24]. Thailand does not need to reinvent the wheel. It needs to scale what already works.

The key ingredients in these models are not financial. They include leadership commitment, data accessibility, and protected space for review and decision-making. The suburban-capital hospital had all three. Participants from other sites frequently noted that without these supports, registry participation felt like a burdensome task rather than a meaningful tool. National leaders should prioritize mechanisms to replicate and scale this kind of success, including training EMS personnel in data literacy and enabling hospitals to integrate OHCA review into their routine operations.

### Structural barriers to scale

The most consistent challenge across all sites was systemic fragmentation. Thailand’s EMS system spans multiple ministries and reporting chains, with weak horizontal integration. The Ministry of Public Health oversees most ALS services and dispatch centers, while the Ministry of Defense and local municipalities operate parallel BLS services. This split governance structure creates administrative silos, data incompatibility, and inconsistent protocol enforcement. Interviewees described situations where different teams arrived at the same scene with different equipment, expectations, and levels of training.

Fragmentation also exists in how data are managed. EMS and hospital teams often use separate platforms, or even paper systems, that do not communicate with each other. At one site, data officers manually reconciled patient IDs from dispatch records with admission logs by phone. In such settings, the idea of tracking 30-day or neurological outcomes is logistically impossible.

This disconnection is not unique to Thailand. Many LMICs face similar challenges, particularly where decentralization has outpaced infrastructure development [25]. However, solutions exist. Brazil’s SAMU system, for example, integrates regional EMS under a centralized national protocol, while Indonesia is piloting linked pre- and in-hospital registries in select cities [26, 27]. What these cases share is national leadership – an agency or ministry responsible for coordinating standards, supporting digital infrastructure, and holding providers accountable.

In Thailand, formal comparisons with non-PAROS sites were not simple, as standardized OHCA data are not available outside participating centers. This reflects a broader systemic challenge: the absence of a consistent national data infrastructure constrains both comparison and coordinated evaluation of OHCA care. While PAROS provides a structured framework for data collection, its current implementation remains limited to selected sites and does not constitute a national standard. Broader adoption is likely shaped by variability in local infrastructure, staffing capacity, documentation practices, and the lack of a nationally mandated OHCA reporting system. Together, these factors suggest that scaling data systems requires not only technical solutions, but also institutional alignment and sustained policy support.

### Toward a national OHCA data system

Based on these findings, we propose a multi-step roadmap for developing a national OHCA registry in Thailand:

1. **Define a core dataset** that includes pre-hospital times, bystander interventions, ROSC, and neurological outcomes. Start small but make these indicators mandatory for reimbursement or performance evaluation.
2. **Build interoperability** between EMS and hospital record systems, starting with shared patient identifiers and automated data exchange platforms. Even partial linkage (e.g., EMS and ER with IPD departments) can improve feedback loops. Implementation should be supported by appropriate data governance frameworks to ensure patient privacy and confidentiality while enabling secure data sharing across systems.
3. **Incentivize participation** through funding mechanisms, such as tying registry completeness to hospital or EMS unit funding, as used in trauma systems globally.
4. **Establish governance** via a national oversight committee with representation from MoPH, NIEMS, academic centers, and local government units. This group should set standards, monitor compliance, and publish national reports.
5. **Train and retain data officers** at regional and hospital levels to support implementation, troubleshoot gaps, and build institutional memory.
6. **Engage communities** through publicly available dashboards, CPR campaigns, and co-developed AED placement strategies. Civil society engagement improves political will and sustainability.

Many of the challenges identified in this study are not unique to Thailand. Similar patterns have been observed in other low- and middle-income countries (LMICs), where EMS systems often face fragmented governance, limited interoperability between pre-hospital and hospital care, and incomplete outcome tracking. Previous studies in LMIC settings have highlighted comparable barriers to registry implementation and system-level coordination, suggesting that these issues reflect systemic constraints rather than context-specific limitations [28, 29].

Because data fragmentation poses a universal challenge across these settings, this proposed roadmap is not exclusive to Thailand; rather, it provides valuable insights applicable to other LMIC contexts. The suggested solutions, particularly unified data governance and defining actionable core datasets while enabling data interoperability among stakeholders, can serve as a practical template for other LMICs seeking to consolidate fragmented OHCA data or broader emergency care data systems.

Within this broader landscape, Thailand is well positioned to lead such reforms. It has an established EMS infrastructure, technical expertise through PAROS participation, and local examples of success. What remains is the political alignment to connect these elements into a cohesive national system.

### Limitations

This study has several limitations. First, it is based on data from three hospitals participating in the PAROS network and may not capture variation across non-participating systems in Thailand. Although these sites include large and relatively well-resourced public hospitals, findings may not reflect variation in smaller, rural, or less-resourced settings. As PAROS participation is limited and not nationally representative, the findings should be interpreted as illustrative of system-level challenges rather than comprehensive of all EMS contexts in the country.

Second, while qualitative interviews were extensive, some stakeholder groups, such as funders and insurance providers, were underrepresented. Third, registry data quality varied across sites, reflecting differences in data collection protocols, staff responsibilities, and institutional emphasis on certain outcomes.

Despite these limitations, the convergence of qualitative and quantitative findings across diverse settings strengthens the transferability of our conclusions. The barriers described here appear to be structural rather than site-specific, and the proposed recommendations align with international best practices.

## Conclusion

Out-of-hospital cardiac arrest (OHCA) remains a significant public health challenge in Thailand, with important gaps in data infrastructure, system integration, and outcome tracking. This study highlights both the potential for improvement through local data-driven initiatives, and the limitations posed by fragmented systems and inconsistent data practices. Strengthening national coordination, improving interoperability between EMS and hospital systems, and standardizing outcome measurements may support more effective and equitable OHCA care. These findings provide a foundation for future research and policy development aimed at improving resuscitation outcomes in Thailand and similar settings.

## Supplementary Information

Below is the link to the electronic supplementary material.

Supplementary Material 1

Supplementary Material 2

Supplementary Material 3

## Data Availability

The datasets generated and/or analyzed during the current study are not publicly available due to confidentiality consent with participants, which restricts sharing of sensitive personal data. However, the data can be made available from the corresponding author upon reasonable request, subject to institutional approval and ethical committee compliance.
