# Supplementary material for: Out-of-hospital cardiac arrest in three EMS centers in northern and central Thailand: a mixed-methods call for a national data infrastructure
Source: BMC Emerg Med. 2026 May 29;26:208. doi: 10.1186/s12873-026-01629-1 (PMC13439877; doi:10.1186/s12873-026-01629-1)
Supplement: Supplementary file 1 [file 12873_2026_1629_MOESM1_ESM.docx]

**Supplementary Appendix 1. Original Thai-language quotes corresponding to Tables 2–6 and in-text excerpts in the Results section**

This appendix provides the original Thai-language versions of all interview excerpts presented in English in the main manuscript, including those appearing in Tables 2–6 and in-text quotations within the Results section.

**Table A1. Thai-language quotes corresponding to Table 2**

| **Quotes** | **Interviewees** |
| --- | --- |
| Neurological outcome data น้อย เพราะ เคส survival to admit ของเรา มันมีไม่เยอะ มันมีส่วนที่ dead at scene แล้วก็ dead ที่ห้องฉุกเฉิน แล้วก็ dead ที่โรงพยาบาล ส่วนที่ survive to discharge เนี่ย มีไม่เยอะ ถ้าจำไม่ผิดของราชวิถี [urban-capital site] มีซัก 6-7% แต่ survive with good neurological outcome มันก็จะเป็น subset ของกลุ่มนี้ เราก็เลยไม่ค่อยเห็นมันขึ้นมา พอเรา export PAROS data ออกมา กลุ่มนี้มันเลยเหลืออยู่นิดเดียว  We have little data on neurological outcomes because there aren’t many cases that survive. A lot of patients are dead on scene, at the ER, or in the hospital. Not many survive until discharge. If I remember correctly, survivors only make up 6-7% at our [urban-capital] hospital, and those who survive with good neurological outcome is a subset of this group, so we barely see them. When we export PAROS data, this group is really small. | EMS Supervisor, Urban Hospital |
| เราไม่ได้เก็บ neurological data แบบจริงๆ จังๆ ไม่ได้มีโทรตามสัมภาษณ์หลังกลับบ้าน ก็มีคนแซวเหมือนกันว่า เราก็น่าจะเก็บซักหน่อยมั้ย เพราะว่ามีคนรอดกลับบ้านก็แค่หลักหน่วยอยู่แล้วต่อปี จริงๆ คนที่รอดกลับไปมันมี set คำถาม แต่เราไม่ได้ให้เค้ามานั่งดูแล้วก็ตอบ บางทีสัมภาษณ์เป็นครึ่งชั่วโมงอะ กว่าจะได้ข้อมูล มันซับซ้อน จริงๆ ควรจะนัดมา follow-up แล้วมีการพูดคุยกันจริงๆ จังๆ อย่าง Singapore เค้าทำเป็น Survival Club เลย (…) ตอนนี้ต้องบอกของไทยเรา เรื่อง quality of life เอาไว้ทีหลัง ตอนนี้ขอให้เอาคนไข้ให้รอดก่อน  We aren’t rigorous with collecting neurological [outcome] data; we don’t call patients to interview after they’ve been discharged. Someone had made a suggestion, too, to start collecting these data because there’re less than 10 patients who can go home alive per year. We have a set of questions for these survivors, but we don’t ask them these questions formally. It would take close to half an hour to collect all those data, it’s complicated. If we wanted to stick to the book, we should make an appointment to do a follow-up with them. Like in Singapore, they even organize a Survivor Club (…) But I have to admit that in Thailand, we can’t focus on quality of life yet, we must prioritize just survival first [regardless of quality]. | Emergency Physician, Suburban Hospital |

**Table A2. Thai-language quotes corresponding to Table 3**

| **Quotes** | **Interviewees** |
| --- | --- |
| ตอนนั้น bystander CPR ผม 20% เอง มันก็เลยเป็นที่มาว่าเราก็เลย attack community CPR จำนวนมากระดับนึง ตอนนี้ขึ้นมา 50 กว่าเปอร์เซ็นต์ละ จาก 20% มันก็อิมแพคค่อนข้างเยอะเหมือนกัน แล้วก็เนี่ยมามี project อื่นๆ ตามมาเรื่อยๆ แล้วทุกๆ project มันก็เริ่มจาก data เนี่ยแหละครับ  Back then, our bystander CPR rate was only 20%, that’s why we tackled the community CPR challenge. Right now we’re up to 50% with quite a large [positive] impact. Since then, we continue to develop other projects. All of them started from having data. | Emergency physician, Suburban-Capital Hospital |
| เราใช้ข้อมูลจาก registry มาดูว่าแต่ละพื้นที่ขาดอะไร แล้วเอาไปปรับตำแหน่ง AED  We gained information from [OHCA] registry and we analyzed to see which areas were missing what. Then we adjusted the locations of AED. | Hospital Admin, Suburban-Capital Hospital |
| เราประชุมทุกอาทิตย์เพื่อดูข้อมูลว่า dispatch ช้าไหม มีที่ไหนควรปรับปรุง  We have a meeting every week to see if our dispatch units are slow or whether there’s anything they should improve on. | EMS Supervisor, Suburban-Capital Hospital |
| เราใช้ telemedicine ช่วยให้เราติดตามและบันทึกข้อมูลได้ดีขึ้น  We use telemedicine to follow and record the data better. | Data Coordinator, Suburban Hospital |
| เรื่อง registry มันก็เป็นพื้นฐานทั้งหมดก่อนแล้วกันครับ พอทำ registry เราก็เจอปัญหาว่า ตัว survival rate เราไม่ดี เราก็เลยมี intervention หลายๆ อย่างมากมาย แล้วเราก็ติดตามด้วย registry  The registry is the foundation of everything. When we started to carry out the registry, we found that our problem was that survival rate wasn’t good. So we initiated many interventions, and we then followed [their results] using the registry. | EMS Supervisor, Suburban-Capital Hospital |

**Table A3. Thai-language quotes corresponding to Table 4**

| **Quotes** | **Interviewees** |
| --- | --- |
| เจ้าหน้าที่ที่เป็นหน่วย service กับศูนย์ที่รับแจ้งน่ะครับ เขาไม่ได้เชื่อใจกัน  The staff members of the service [EMS] units and of the emergency call centres don’t trust one another. | EMS Leader, Urban Hospital |
| ALS จะไม่ออกจากฐานถ้า BLS ยังไม่ confirm ว่าเป็นเคสจริง  The ALS team will not leave their base if the BLS team hasn’t confirmed that the case is real [qualified for Advanced aid] yet. | Dispatcher, Urban Hospital |
| แต่ละที่มีระบบของตัวเอง บางที่ก็ไม่ให้ BLS ย้ายผู้ป่วย ถ้าไม่ใช่หน่วยเดียวกัน  Each place [hospital/dispatch centre] has their own system. Some places don’t approve the BLS team to move the patient if that BLS team isn’t of their own network. | BLS Team Member, Regional Hospital |
| ตอนมาถึงเคส บางที BLS กับ ALS ก็เกี่ยงกันเรื่องใครจะรับผิดชอบ  When they arrive at the scene, sometimes the BLS and ALS teams would keep passing the buck about who should be responsible [for the patient]. | ALS Paramedic, Urban Hospital |

**Table A4. Thai-language quotes corresponding to Table 5**

| **Quotes** | **Interviewees** |
| --- | --- |
| เราตามได้แค่ ER [...] พอขึ้นวอร์ดปุ๊บ เราตามไม่ได้แล้ว  We can only keep track [of the data] within the ER service. When the patient is admitted as an in-patient, we cannot keep following their data. | Data Officer, Regional Hospital |
| เราไม่มีระบบที่เชื่อมโยงจาก EMS ไปยังโรงพยาบาลได้แบบอัตโนมัติ  We don’t have the system that automatically connects the EMS [data pool] with the hospital. | IT/Registry Staff, Suburban Hospital |
| ข้อมูลที่ EMS บันทึกไว้ไม่ได้ถูกเอาไปใช้ต่อในโรงพยาบาล  The data that the EMS team records don’t get used within the hospital. | Hospital Admin, Urban Hospital |
| อย่าง chart ในวอร์ดเนี่ย บางทีมันยังไม่เป็น electronic 100% บางทีต้องไปขอเวชระเบียนมา review ก็จะยังมีข้อจำกัดอยู่ เพราะว่า สิทธิ์การเข้าถึงข้อมูลของฝั่ง in-patient น่ะ มันก็จะยังไม่ได้รายละเอียดเท่าไหร่ คนหน้างานจะไม่ได้สิทธิ์ในการดู record ของการรักษาไปถึง final discharge บางทีอาจจะได้ discharge summary ซักนิดหน่อย อันนี้มันก็ขึ้นอยู่กับความละเอียดของตัว discharge summary ว่าเค้าเขียนมาให้เราละเอียดแค่ไหน  Some inpatient charts aren’t 100% electronic, sometimes we have to ask the record department for specific data to do a case review. So that’s a limitation. Our clearance to access data from the in-patient departments isn’t high. The front-line workers [EMS personnels] do not have the clearance to view treatment records until discharge. In some cases, we might obtain a discharge summary. But it all depends on luck whether we get a detailed discharge summary or not. | Emergency Physician, Urban Hospital |
| บางทีเราไม่มีใครตามไปประเมิน ยกเว้นว่าช่วงไหนที่เรามีคนที่ตามเก็บให้เราถี่มากๆ อันนี้เราอาจจะยังพอเห็นภาพว่าระหว่างที่คนไข้ admit อยู่ช่วง 1-2 สัปดาห์เนี่ย เค้าเป็นยังไง เรายังเดินตามไปดูที่วอร์ดได้ ไปดูตรงนั้นเลย แต่ถ้าเป็น 3 เดือนตามทีอย่างเนี้ย มันไม่เห็นอะไร (…) มันต้องเชื่อม pre-hospital เชื่อมเข้ากับข้อมูลที่เป็น emergency [ER] แล้วก็เชื่อมกับฐานข้อมูลที่เป็น in-patient ในโรงพยาบาล ข้อมูล 3 phase ถ้ามีทีมใหญ่จริง มันเป็นไปได้ที่จะตามข้อมูลจุดนี้  Sometimes we don't have anyone to follow up for evaluation, except during periods when we have someone collecting data for us very frequently. In this case, we can still gain an understanding of the patient's condition during the 1-2 weeks they are admitted. We can still go and monitor them in the ward, but if it's a 3-month follow-up, we don't see anything. (…) It needs to connect pre-hospital data with emergency [ER] data and then with in-patient data in the hospital. If there is a large team, it is possible to follow up on this data point. | Emergency Physician, Urban Hospital |

**Table A5. Thai-language quotes corresponding to Table 6**

| **Quotes** | **Interviewees** |
| --- | --- |
| ศูนย์เอราวัณน่ะ มันเป็นหน่วยของท้องถิ่น (...) policy เขาจะเปลี่ยนตามผู้บริหาร  The Erawan Centre [Bangkok Emergency Medical Centre] is a local administrative organization’s unit. (…) Their policy changes according to their leaders. | Hospital Admin, Urban Hospital |
| ไม่มีหน่วยงานไหนรับผิดชอบเรื่อง OHCA โดยตรงแบบชัดเจน  There’s no agency that’s directly and clearly responsible for the OHCA patients. | NIEMS Official |
| นโยบายแต่ละพื้นที่ไม่เหมือนกัน dispatch บางที่ใช้เวลาเยอะกว่าจะยืนยัน  The policies differ in every area. Some dispatch units spend a lot of time before they commit [to cases]. | Dispatcher, Regional Hospital |
| ระบบของกรุงเทพกับต่างจังหวัดใช้คนละแบบเลย จะรวมข้อมูลกันก็ลำบาก  The systems in Bangkok and in provinces outside of Bangkok are completely different. It’s difficult to compile all the data. | IT Admin, Urban Hospital |

**Abbreviation:** NIEMS, National Institute for Emergency Medicine.

**Table A6. Thai-language quote corresponding to in-text excerpt in Section 3.1**

| **Quotes** | **Interviewees** |
| --- | --- |
| พอมี data แล้ว เขาก็จะไปคุยกับผู้บริหารว่า โอเค เขาจะทำระบบนี้ ตัวเลข report เราเป็นอย่างนี้ อยากจะทาให้ดีขึ้น (…) เป็นการทำเคสรีวิวน่ะครับ ทำเป็นเรื่องปกติทุกสัปดาห์เลย  We use the data to communicate with the hospital’s board what we want to do. Like, “These are our numbers from the reports; we want to improve them.” (…) They [the numbers] are from reviewing our cases; we do that every week. | Paramedic, Suburban-Capital Hospital |

**Table A7. Thai-language quote corresponding to in-text excerpt in Section 3.3**

| **Quotes** | **Interviewees** |
| --- | --- |
| ถ้ามันมีกระแสสังคมอยู่น่ะ ผู้บริหารก็จะเอาละ จะขอข้อมูล จะขอความเห็นภายใน 1 วัน ว่าจะให้ออกตอบ public ยังไง  If there is public momentum, senior leadership may suddenly request information in order to respond to the media, often requiring a response within one day. | Health Policy Advisor |
