# Supplementary material for: Out-of-hospital cardiac arrest in three EMS centers in northern and central Thailand: a mixed-methods call for a national data infrastructure
Source: BMC Emerg Med. 2026 May 29;26:208. doi: 10.1186/s12873-026-01629-1 (PMC13439877; doi:10.1186/s12873-026-01629-1)
Supplement: Supplementary file 2 [file 12873_2026_1629_MOESM2_ESM.docx]

# **Supplementary Materials**

**Supplementary Figure 1:** Study flow diagram

Of 3,050 OHCA cases recorded in the Thai PAROS registry between 2017 and 2023, 791 were excluded due to non-EMS transport, traumatic etiology, pediatric age, or missing timestamp data. The final analysis included 2,259 adult non-traumatic OHCA patients who received EMS care across the three study hospitals.

**Supplementary Table 1.** Patient and Arrest Characteristics

| **Patient Characteristics** | | **Thailand OHCA Data Collection Centres** **(Total = 2259)** | | | ***P-value*** |
| --- | --- | --- | --- | --- | --- |
|  |  | **Regional** | **Urban-capital** | **Suburban-capital** |  |
|  |  | **(2017-2023)** | **(2017-2020)** | **(2017-2023)** |  |
|  |  | **(n = 569)** | **(n = 785)** | **(n = 905)** |  |
|  |  | N (%) | N (%) | N (%) |  |
| Age (year), mean ± SD | | 57.0 ^a^ ±21.4 | 61.8 ^b^ ±19.4 | 66.0 ^c^ ±17.1 | <0.001 |
| Male | | 380 (66.8) | 512 (65.2) | 547 (60.4) | 0.026 |
| Ethnicity | Thai | 490 (86.1) | 271 (34.5) | 896 (99.1) | <0.001 |
|  | Other | 79 (13.9) | 514 (65.5) | 9 (0.9) |  |
| Co-morbidity | Heart disease | 262 (46.1) | 221 (26.9) | 131 (14.5) | <0.001 |
|  | Diabetes Mellitus | 86 (15.1) | 169 (21.5) | 253 (28) | <0.001 |
|  | Cancer | 30 (5.3) | 75 (9.6) | 91 (10.1) | 0.004 |
|  | Hypertension | 140 (24.6) | 237 (30.2) | 343 (37.9) | <0.001 |
|  | Renal disease | 55 (9.7) | 72 (9.2) | 88 (9.7) | 0.919 |
|  | Respiratory disease | 40 (7) | 61 (7.8) | 53 (5.9) | 0.289 |
|  | Dyslipidemia | 45 (7.9) | 39 (4.9) | 75 (8.3) | 0.019 |
|  | Stroke | 28 (4.9) | 47 (5.9) | 50 (5.5) | 0.698 |
|  | HIV | 5 (0.9) | 7 (0.9) | 5 (0.6) | 0.667 |
|  | Other | 88 (15.5) | 119 (15.2) | 143 (15.8) | 0.936 |
| Estimated CCI, median (IQR) | | 3 (1-4) | 3 ^b^ (1-5) | 3 ^c^ (2-5) | <0.001 |
| Location type | Home Residence | 312 (54.8) | 633 (80.6) | 767 (87.6) | <0.001 |
|  | Street/Highway | 103 (18.1) | 63 (7.9) | 43 (4.8) |  |
|  | Public/Commercial Building | 100 (17.6) | 57 (7.3) | 34 (4.1) |  |
|  | Healthcare Facility | 24 (4.2) | 15 (1.9) | 10 (1.1) |  |
|  | Other | 30 (5.3) | 17 (2.2) | 48 (5.3) |  |
| Cause of arrest | Presumed Cardiac Aetiology | 205 (36.0) | 240 (30.6) | 249 (27.5) | <0.001 |
|  | Respiratory | 180 (31.6) | 48 (6.1) | 133 (14.7) |  |
|  | Others | 25 (4.4) | 44 (5.6) | 124 (13.7) |  |
|  | Unknown | 159 (27.9) | 453 (57.7) | 399 (44.1) |  |
| First arrest rhythm | Asystole | 176 (30.9) | 576 (73.4) | 634 (70.1) | <0.001 |
|  | PEA | 101 (17.8) | 86 (10.9) | 136 (15.0) |  |
|  | Unknown unshockable rhythm | 201 (35.3) | 32 (4.1) | 41 (4.5) |  |
|  | VF | 51 (8.9) | 62 (7.9) | 83 (9.2) |  |
|  | VT | 5 (0.9) | 1 (0.1) | 3 (0.3) |  |
|  | Unknown shockable rhythm | 19 (3.3) | 8 (1.0) | 3 (0.3) |  |
|  | Unknown | 201 (35.3) | 32 (4.1) | 41 (4.5) |  |
| Witnessed arrest | Bystander – Family | 125 (21.8) | 297 (37.8) | 423 (46.7) | <0.001 |
|  | Bystander – Healthcare provider | 30 (5.3) | 58 (7.4) | 42 (4.6) |  |
|  | Bystander – Lay person | 71 (12.5) | 66 (8.4) | 115 (12.7) |  |
|  | EMS or Private ambulance | 23 (4)  320 (56.2) | 21 (2.7) | 57 (6.3) |  |
|  | Not witnessed |  | 343 (43.7) | 268 (29.6) |  |

**Abbreviations:** CCI, Charlson Co-morbidity Index; EMS, Emergency Medical Service; PEA, Pulseless Electrical Activity; VF, Ventricular Fibrillation; VT, Ventricular Tachycardia; Significant differences from post-hoc pairwise comparisons (*p* < 0.05) using Bonferroni method: ^a^ regional vs. urban-capital, ^b^ urban-capital vs. suburban-capital, ^c^ suburban-capital vs. regional

Of the 2,259 adult non-traumatic OHCA patients included in the analysis, baseline demographic and arrest characteristics are presented in Supplementary Table 1. Significant differences were observed across the three study hospitals in age distribution, ethnicity, comorbidity profiles, arrest location, presumed etiology, initial rhythm, and witness type.

**Supplementary Table 2.** Detailed neurological outcome (CPC/OPC) data across study sites

| **Patient Characteristics** | **Thailand OHCA Data Collection Centers**  **(Total = 2259)** | | | | | | ***P-value*** |
| --- | --- | --- | --- | --- | --- | --- | --- |
|  | **Regional hospital**  **(2017-2023)**  **(n = 569)** | | **Urban-capital hospital**  **(2017-2020)**  **(n = 785)** | | **Suburban-capital hospital**  **(2017-2023)**  **(n = 905)** | |  |
|  | N | (%) | N | (%) | N | (%) |  |
| Patient neurological status – Overall Performance Category |  |  |  |  |  |  |  |
| OPC1 | 1 | 0.65 | 0 | 0 | 10 | 9.62 | <0.001 |
| OPC2 | 0 | 0 | 0 | 0 | 4 | 3.85 |  |
| OPC3 | 0 | 0 | 0 | 0 | 1 | 0.96 |  |
| OPC4 | 0 | 0 | 0 | 0 | 1 | 0.96 |  |
| Patient neurological status – Cerebral Performance Category |  |  |  |  |  |  |  |
| CPC1 | 1 | 0.65 | 0 | 0 | 10 | 9.62 | <0.001 |
| CPC2 | 0 | 0 | 0 | 0 | 6 | 1.36 |  |
| CPC3 | 0 | 0 | 0 | 0 | 1 | 0.96 |  |
| CPC4 | 0 | 0 | 1 | 0.54 | 3 | 2.88 |  |
| Patient Neurological Status |  |  |  |  |  |  |  |
| Collected | 0 | 0.00 | 1 | 0.13 | 10 | 1.10 | <0.001 |
| Not Collected | 569 | 100 | 784 | 99.87 | 895 | 98.90 |  |
| 30-day Post-arrest Hospital Outcome |  |  |  |  |  |  |  |
| Alive | 36 | 6.33 | 12 | 1.53 | 19 | 2.1 | <0.001 |
| Death | 533 | 93.67 | 773 | 98.47 | 886 | 97.9 |  |

**Abbreviations:** CPC, Cerebral Performance Category; CCI, Charlson Co-morbidity Index; OCP, Overall Performance Category; Significant differences from post-hoc pairwise comparisons (p < 0.05) using Bonferroni method: a regional vs. urban-capital, ^b^ urban-capital vs. suburban-capital, ^c^ suburban-capital vs. regional.
