# Supplementary material for: Out-of-hospital cardiac arrest in three EMS centers in northern and central Thailand: a mixed-methods call for a national data infrastructure
Source: BMC Emerg Med. 2026 May 29;26:208. doi: 10.1186/s12873-026-01629-1 (PMC13439877; doi:10.1186/s12873-026-01629-1)
Supplement: Supplementary file 3 [file 12873_2026_1629_MOESM3_ESM.docx]

**Interview Guide for National OHCA Data Collection Program in Thailand**

1. **Introduction**

- Introduction of the interviewer and explanation of the study purpose.
- Assurance of confidentiality and voluntary participation.
- Confirmation of consent to record for accuracy.

2. **Current Understanding and Involvement**

- Can you describe your current involvement with data collection regarding OHCA?
- **Present:** How is this data utilized within your institution?
  - **Past**: Without this data set, how does your institution handle these activities effectively? What other tools do you use to fulfill these purposes? (What is this dataset replacing/enhancing?)
  - **Vision**: Are there new innovations or tasks that your institution can initiate from this data set? (new innovation/benefits outside of current capacity)
  - **Future:** If and when this project is fully developed and to be adopted nationally, how do you think your/other institution(s) can benefit from the data set?

3. **PAROS questionnaire for OHCA data collection – for respondents who have directly handled the questionnaire**

- For you, what are the most important factors that help OHCA patients survive in Thailand? Do you think PAROS is covering that?
- General comments: What do you think about the PAROS questionnaire quality? What are the good parts? What is it still missing?
- What are your opinions about the preliminary results? Were you surprised by them?
- How do you think this data can be used outside of the hospital context?

4. **Support for a National Data Collection Program**

- **Institutional**: What are your thoughts on implementing a national OHCA data collection program in Thailand?
  - **Personal**: How did you feel when you learned that this data collection project was going to take place at your institution? (e.g. Was it too much work or were you excited because it’s something you could use to fill the current gaps?)
- Would you support the establishment of such a program? Why or why not?
  - What supports are lacking to successfully adopt the program at your institution?
  - Who/what institutions need to be a strong support for this program to happen and how?
  - If it is not your institution, what are the appropriate institutions/personnel to do it?

5. **Anticipating Challenges**

- What challenges do you anticipate in rolling out a national OHCA data collection program?
  - How should these challenges be resolved?
  - **Depersonalize question**: If the staff in your institution or the past/future personnel or your position to be against this program, what do you think their reasons are?
- (Probe if not already covered) Are there any specific logistical, technological, or financial barriers you foresee?
  - Do you think the resources that your institution or the national management body uses to collect and utilize this dataset worth the benefits of it?
  - Should you employ the limited resources on something else for similar benefits?
  - (Probe if time permits) Do the pros outweigh the cons?

6. **Planning a Pilot Program**

- How would you design a pilot program for national OHCA data collection? What key elements would you include?
- Which regions or hospitals would be ideal for initiating a pilot data collection program and why?
- How would you determine the success of the program? What elements would you count?
- What steps should be taken to scale the pilot program to a national level after the initial phase?

7. **Stakeholder Roles and Collaboration**

- What roles should different stakeholders (e.g., local health departments, emergency services, hospitals) play in the pilot program?
- How can we ensure effective collaboration and communication among stakeholders in the pilot program?
- What are the factors that can impact the success of this program? (e.g. human, technological, financial requirements)

8. **Resources and Infrastructure Needs**

- What infrastructure improvements (for examples, institutional collaboration, intra-communication, education, recreation, public spaces, access to basic living needs, etc.) are necessary for the pilot program to succeed?
- What resources (human, technological, financial) are needed to start and sustain the pilot program?

9. **Impact and Long-term Goals**

- What long-term impacts do you hope to achieve with the national OHCA data collection program?
- Do you think this project is something Thailand’s public health sector should invest in? If not, what other areas should it focus on?
- How should the program evolve to meet future healthcare challenges and technological advances?

10. **Policy and Regulation**

- What policy support is needed to facilitate the pilot and eventually the national rollout?
- How can this initiative align with existing health policies and data protection laws?

11. **Additional Feedback**

- Are there any other features or support mechanisms that would be crucial for the success of the pilot program?
- Is there anything else you would like to add that hasn't been covered?

12. **Closing**

- Summarize the discussion and confirm any follow-up actions.
  - Snowball sampling: ask about other contact recommendations who can provide insights to this project implementation.
- Express gratitude for the participant's time and insights.

13. **Follow-Up**

- Provide contact information for further communication.
- Outline how and when the interview findings will be shared.
